# Effect of Active and Passive Smoking on Retinal Nerve Fibre Layer and Ganglion Cell Complex

**DOI:** 10.1155/2017/6354025

**Published:** 2017-04-12

**Authors:** Amany Abd El-Fattah El-Shazly, Yousra Ahmed Thabet Farweez, Lamia Salah Elewa, Yasser Abdelmageuid Elzankalony, Botheina Ahmed Thabet Farweez

**Affiliations:** ^1^Department of Ophthalmology, Ain Shams University, Cairo, Egypt; ^2^Department of Clinical Pathology, Ain Shams University, Cairo, Egypt

## Abstract

*Aim.* To evaluate the possible structural and functional changes in the retinal nerve fibre layer (RNFL) and the ganglion cell complex (GCC) of chronic smokers and compare them with those of passive healthy smokers using spectral domain optical coherence tomography (SD-OCT) and pattern electroretinogram (PERG). *Materials and Methods*. We include 80 active chronic smokers and 80 age- and sex-matched healthy passive smokers. After a full ophthalmological examination, SD-OCT and PERG were tested for all participants. Urinary levels of cotinine and creatinine with subsequent calculation of the cotinine creatinine ratio (CCR). *Results*. Inferior and superior quadrants of RNFL were thinner in group I, but nasal and temporal quadrants did not show significant difference between the groups. There were no significant differences of GCC values between the two groups. There was no significant difference of PERG-P50 amplitude and latency; however, PERG-N95 showed significant difference between the two groups. Multiple regression analyses demonstrated that the number of cigarettes/day, urinary cotinine, and PERG-N95 amplitude are the most important determinants for both superior and inferior RNFL thicknesses. *Conclusion*. RNFL thickness decreases in chronic, healthy, heavy cigarette smokers, and this thinning is related to the number of cigarettes/day, urinary cotinine, and PERG-N95 latency and amplitude.

## 1. Introduction

Several studies have suggested that cigarette smoking, which was proven to be a risk factor for atherosclerotic complications in the aortic, coronary, and cerebral circulation [1], causes damage in other organs through the local metabolic and vascular effects of systemically absorbed products [2]. Tobacco smoke contains oxidizing agents that yield free radicals and may cause cell damage and even cell death by apoptosis [3].

Common eye diseases such as cataract [4], age-related macular degeneration [5], retinal venous occlusion [6], anterior ischemic optic neuropathy [7], thyroid ophthalmopathy [8], and even primary open-angle glaucoma [9] were found to be related to cigarette smoking.

Grzybowski and Holder [10] speculated that tobacco optic neuropathy, characterized by bilateral central visual deterioration, may occur as a result of direct toxic damage to the optic nerve particularly in heavy active smokers.

Past studies reported the alteration in visual fields of healthy heavy cigarette smokers documented by decreased retinal sensitivity and peripheral scotomata in the visual fields [11]. Moreover, the effects of smoking on ocular diseases are significantly dose-dependent risks, and morbidity correlates with the increase in smoking index [7].

The peripapillary retinal nerve fibre layer (RNFL) and ganglion cell-inner plexiform layer complex (GCIPL) thickness, which were proved to be thinned in glaucomatous [12] and nonglaucomatous optic neuropathies [13] and other central nervous diseases [14], can be successfully evaluated with high-definition optical coherence tomography (HD-OCT) devices. Pattern electroretinogram (PERG) has considerable value in detailed evaluation of macular diseases as well [15]. However, to the best of our knowledge, no study has evaluated the effects of chronic smoking on these parameters in healthy subjects.

The aim of this study was to evaluate the possible structural and functional changes in RNFL and GCC of chronic heavy smokers and compare it with those of passive healthy smokers using OCT and PERG.

### 1.1. Subjects and Methods

This cross-sectional study was conducted between January 2015 and May 2016, with the participants recruited from the patients' relatives and hospital staff of the outpatients' clinics of Ain Shams University Hospital. The study adhered to the tenets of the Declaration of Helsinki, and it was approved by the ethical committee of the Faculty of Medicine, Ain Shams University. Informed written consent was obtained from all individual participants before the study began.

A total of 160 participants were enrolled in the study. The participants were classified into two groups; the active smoker group included 80 participants who were healthy cigarette smokers—who smoke at least 10 cigarettes for 10 years—with no other systemic or ocular disease (group I) and the passive smoker group consisted of 80 age- and gender-matched healthy never smoking participants but in close contact with smokers (group II). We could not add a 3rd group (never smoked and without exposure to passive smoking) as the smoking is highly prevalent in our environment. One eye of each participant was included.

### 1.2. Patient Selection

We included participants whose age ranged from 25 to 35 years, spherical refraction between +1.0 and −1.0 diopters, best corrected visual acuity (BCVA) of 20/20 or better, intraocular pressure (IOP) < 22 mmHg, and axial length (AL) < 25 mm. In group II, participants smoke at least 10 cigarettes/day for at least 10 years duration.

We excluded any participant with pre-existing retinal diseases, glaucoma, previous LASIK or retinal surgery, previous eye trauma, a history of alcohol intake, any systemic disease specially diabetes mellitus (DM), uncontrolled hypertension, recurrent migrainous attacks, or a history of medication intake within the last 3 months, especially systemic vasoactive drugs.

### 1.3. Ophthalmological Examination

Full ophthalmological evaluations, including best corrected visual acuity (BCVA) using Snellen charts, slit-lamp biomicroscopy, Goldmann applanation tonometry, and fundus examination, were performed. Then one eye of each participant was subjected to the following investigations.

*Axial length measurement (AL)* was peformed using A-scan ultrasonography (PacScan 300A, Sonomed Escalon Inc., New York, USA). Prior to measurement, the cornea was anesthetized with one drop of topical 0.4% benoxinate HCl. Five readings were obtained to calculate an average value. The standard deviation was set below 0.1 mm for each subject.

*Central corneal thickness (CCC) measurement* was performed using SD-OCT (Retina Scan RS-3000 advance, NIDEK, Gamagori, Japan) by the anterior segment OCT program. We used the single corneal line scan horizontally with the scan width of 6 mm centered on the center of the cornea, in which the single scan was averaged from 10 A-scans to gain the best quality image and least noise. The resolution of the single scan of the 10 A-scans was 1024 points. Then the CCT was measured from the most superficial hyperreflective line representing the corneal epithelium to the deepest hyperreflective line indicating the corneal endothelium.

*Retinal nerve fibre layer (RNFL) measurement* was performed using spectral domain optical coherence tomography (SD-OCT) (Retina Scan RS-3000 advance, NIDEK, Gamagori, Japan). After pupillary dilatation using Mydriacyl 0.5% (Alcon Inc.) eye drops, the peripapillary RNFL was measured by 3 circular scannings around the optic nerve in an area with a diameter of 3.4 mm. We recorded the RNFL thicknesses at the superior, nasal, inferior, and temporal quadrants, and at each clock hour, the mean values were calculated. The RNFL thickness was differentiated from other retinal layers using edge detection algorithm.

*Ganglion cell layer (GCL) thickness map* was performed with SD-OCT. The GCL thickness map was obtained from the macular map scan and calculated automatically by the device. It is composed of eight sectorial thickness measurements in two concentric circles with diameters of 4.5 and 9 mm centered on the fovea. The area bounded by the outer (9 mm) and middle (4.5 mm) circles forms the outer ring (OR), while the area bounded by the middle (4.5 mm) and inner (1.5 mm) circles forms the inner ring (IR). Each ring is divided into superior-temporal, superior-nasal, inferior-temporal, and inferior-nasal quadrants. STIM, SNIM, ITIM, and INIM stand for superior-temporal of the inner macula, superior-nasal of the inner macula, inferior-temporal of the inner macula, and inferior-nasal of the inner macula, respectively. STOM, SNOM, ITOM, and INOM stand for superior-temporal of the outer macula, superior-nasal of the outer macula, inferior-temporal of the outer macula, and inferior-nasal of the outer macula, respectively. The central 1.5 mm circle centered on the macula, being devoid of ganglion cells, has no measurement.

Also, the central 9 mm circle centered on the fovea is divided into two halves: upper and lower halves with the total GCL thickness measured all over each half.

*Pattern electroretinogram (PERG)* was recorded with the RETI-port/scan 21 (Roland Consult, Brandenburg, Germany) using an implemented protocol in the system software. Binocular stimulation was used with suitable correction of any refractive error in relative with the eye-screen distance. No dilation of the pupils was required with the PERG to maximize the retinal image quality, and central fixation was determined (patient was monitored with a TV camera). Parameters of the PERG stimulation were the following: 21″ CRT monitor with a frame rate equal to 75 fps was used; black-and-white backward-moving checkerboard (30° field of view (FOV)) was exposed to the patient, with a check size equals to 1° 2′; temporal frequency of 4.0 ± 8.0 reversal rate (rps) is 2.0 ± 0.40 Hz; Michelson contrast is 97%; and luminance for white elements was 120 candelas per square meter (cd/m^2^).

The electrodes placed were the following: the ground-gold disk electrode over the forehead, the HK-loop electrode into the lower fornix, and the surface reference electrodes on the skin near the ipsilateral outer canthus of each eye.

Parameters of the recording system were as follows: amplifier sensitivity is 20 microvolts (*μ*V)/division filters, filters of frequency is 1–100 Hz, artifact discard threshold was 95% (for the amplifier range ± 100 *μ*V), notch filters were off, averaging of 200 sweeps, and sweep time was 250 ms (time base: 25 ms/division). Two successive waveforms were recorded; then, they were off-line being an average and interpreted. The PERG test parameters were in accordance with the ISCEV standard [16].

### 1.4. Urine Sample Collection

Urine samples were collected in aseptic plastic containers to measure urinary levels of cotinine [17] and creatinine [18], with subsequent mathematical calculation of the cotinine creatinine ratio (CCR), and then supernatant was carefully collected after centrifugation for 20 minutes at 2000–3000 rpm. The supernatant was used to measure urinary levels of cotinine and creatinine with subsequent mathematical calculation of the cotinine creatinine ratio (CCR).

Urinary cotinine level was determined by enzyme-linked immunosorbent assay (ELISA) (Sunlong Biotech Co. LTD., Catalog number SL0528Hu) in the patients and control groups according to the manufacturer's instructions. A solid phase ELISA was employed based on the basic principle of a sandwich ELISA. The micro-ELISA strip plate wells are precoated with an antibody specific to cotinine. Samples are added to the wells and combined to the specific antibody. Then, a horseradish peroxidase- (HRP-) conjugated antibody specific for cotinine is added to each well and incubated. Free components are washed away. The TMB substrate solution is added to each well. Only those wells that contain cotinine and HRP-conjugated cotinine antibody will appear blue in color and then turn yellow after the addition of the stop solution. The optical density (OD) is measured spectrophotometrically at wavelength of 450 nm. The OD value is proportional to the concentration of cotinine.

Urinary creatinine level was measured by the Jaffe kinetic reaction with picric acid (ADVIA Chemistry, Siemens Healthcare Diagnostics, NY, USA).

The cotinine creatinine ratio (CCR) of each sample was calculated from the equation CCR = urinary  cotinine  (ng/ml)/urinary  creatinine  (ng/mg).

### 1.5. Statistical Analysis

We analyzed data using the Statistica software, version 10. Quantitative variables were expressed as mean ± SD. Descriptive statistics were done using chi-square analysis. An independent sample *t*-test and Pearson's correlation analysis were used for the statistical analysis of the data. Regression analyses were done to assess the different factors that can affect the RNFL thickness. Significance was evaluated at *p* < 0.05 levels.

## 2. Results

The study included 80 participants (80 eyes) in each group; they were 75 males (93.75%) and 5 females (6.25%) in the active smoker group and 74 males (92.5%) and 6 females (7.50%) in the passive smoker group (Table 1). Their ages ranged from 20 to 35 years.

There were no statistical significant differences between the two groups regarding age, visual acuity (VA), BCVA, spherical equivalent (SE), intraocular pressure (IOP), axial length (AL), and central corneal thickness (CCT) (Table 2).

The active smokers had smoked an average of 23.50 ± 9.69 cigarettes per day (range, 10–60). The mean smoking duration of the active smokers was 19.05 ± 4.89 years (range, 10–30 years) (Table 2).

An independent *t*-test showed significant differences between the two groups for urinary cotinine (*p* < 0.0001) and CCR (*p* < 0.0001) (Table 2).

Inferior and superior quadrants of RNFL were thinner in the active smoker group (*p* < 0.0001 and *p* < 0.0001, resp.), but nasal and temporal quadrants did not show significant difference (*p* = 0.82 and *p* = 0.18, resp.) (Table 3).

There were no significant differences of GCC values between the active smoker group and passive smoker group (Table 4).

There was no significant difference of PERG-P50 amplitude or latency; however, PERG-N95 amplitude and latency showed statistical significant difference between the active smoker group and passive smoker group (Table 5).

Both superior and inferior RNFL thicknesses showed significant negative correlation with the number of cigarettes/day, urinary cotinine, and PERG-N95 latency (*p* < 0.0001) while showed positive correlation with PERG-N95 amplitude (*p* < 0.0001) (Table 6).

Regression summary for superior RNFL thickness in the active smokers is *R* = 0.87, *R*^2^ = 0.75, adjusted *R*^2^ = 0.72, and *F* (10, 69) = 21.195.

Regression summary for inferior RNFL thickness in the active smokers is *R* = 0.870, *R*^2^ = 0.760, adjusted *R*^2^ = 0.73, and *F* (10, 69) = 21.97.

Multiple regression analyses for the active smoker group show which factor in the study was the most important determinant of superior and inferior RNFL thicknesses. We found that the number of cigarettes/day, urinary cotinine, and PERG-N95 amplitude are the most important determinants for both superior (*β* = −0.85, −0.46, and 0.31 with *p* < 0.0001, <0.0001, and 0.02, resp.) and inferior RNFL thicknesses (*β* = −0.84, −0.53, and 0.30 with *p* < 0.0001, <0.0001, and 0.02, resp.) (Tables 7 and 8).

## 3. Discussion

In the recent years, OCT and PERG have proven to be a reliable technology to assess the peripapillary RNFL and GCC [12, 16].

We conducted this study to evaluate the effects of smoking on GCC and RNFL thickness. Our results showed that average RNFL and GCC were not affected. The analysis of the quadrants revealed that inferior and superior quadrants of RNFL were thinner in the active smokers than those of the passive smokers, but temporal and nasal quadrants were not. This deviates from the results reported by Moschos et al. [19] who found that the GCC was significantly thinner in smokers than in controls.

Although Dervişoğulları et al. [20] stated that the nasal, temporal, and central retinal thickness did not differ significantly between smokers and control groups, they proved that the mean RNFL was significantly thinner in the smokers group compared to that in the controls (inferior and superior quadrants of RNFL decreased in smoker group but temporal and nasal quadrants did not seem to be changed [21], which is in agreement with our reports.

There was no significant difference of PERG-P50 amplitude or latency; however, PERG-N95 showed statistical significant difference between the active smoker group and passive smoker group. Worthy of notice is that PERG-N95 showed significant differences among active smoker and passive smoker groups which are the tests for optic nerve functions. These results might denote that smoking targets the RNFL and optic nerve.

Although Hepsen and Evereklioglu [11] suggested that retinal sensitivity decreases in chronic, healthy, heavy cigarette smokers, based on the automated perimeter results, and Tamaki et al. [22] proved that cigarette smoking caused little decrease in blood tissue velocity and decreased peripheral vessel diameters in the optic nerve head and probably in the choroid in young healthy light smokers, there is no other previous study that evaluated the effect of chronic smoking on PERG.

The results obtained in our testing found that both superior and inferior RNFL thicknesses showed negative correlation with the number of cigarettes/day, urinary cotinine, and PERG-N95 latency while showed positive correlation with PERG-N95 amplitude.

Multiple regression analyses were studied for the active smoker group to show which factor in the study was the most important determinant of superior and inferior RNFL thicknesses. We found that the number of cigarettes/day, urinary cotinine, and PERG-N95 amplitude are the most important determinants for both superior and inferior RNFL thicknesses.

RNFL thickness decreased in chronic heavy smokers, and the thinning is related to the amount of smoking rather than duration; and it could be due to direct neurotoxic effect on the optic nerve and reduced blood flow due to the vasoconstrictive effect of nicotine [23].

Yoshida et al. [24] suggested that smoking might be related to the elevation of IOP in middle-aged subjects; however, in our study, mean IOP values did not show any significant difference between smokers and controls.

There was no correlation between duration of smoking and RNFL thickness. From these results, we concluded that the amount of smoking is more effective than the duration of smoking in RNFL thinning.

The study limitations are the relatively small sample size, multicenter studies may be more valued, and cross-sectional design would not provide further and detailed results.

In conclusion, our study results suggested that RNFL thickness decreases in chronic, healthy, heavy cigarette smokers, and this thinning is related to the number of cigarettes/day, urinary cotinine, and PERG-N95 latency while showed positive correlation with PERG-N95 amplitude.

## Figures and Tables

**Table 1:** Gender distribution in the two studied groups.

Active smokers		Passive smokers	
Males	Females	Males	Females
75	5	74	6

Chi-square (*χ*^2^)		*p* value	

0.10		0.75	

**Table 2:** Demographical and clinical characteristics of the active smokers and passive smokers.

Variable	Active smokers	Passive smokers	*t* value	*p*
	Mean ± SD	Mean ± SD		
Age (years)	26.28 ± 3.83	25.53 ± 3.90	1.22	0.23
VA (logMAR)	0.05 ± 0.09 (20/40–20/20)	0.05 ± 0.08 (20/40–20/20)	0.08	0.93
BCVA (logMAR)	0.00 ± 0.00 (20/20–20/20)	0.00 ± 0.00 (20/20–20/20)	—	—
SE (diopters)	−0.36 ± 0.41	−0.37 ± 0.28	0.23	0.82
AL (mm)	24.18 ± 0.30	24.22 ± 0.26	−0.83	0.41
IOP (mmHg)	13.13 ± 1.14	13.05 ± 1.08	0.49	0.62
CCT (mm)	544.76 ± 10.11	542.86 ± 12.35	1.06	0.29
Urinary cotinine (ng/ml)	511.58 ± 152.23	41.65 ± 9.92	27.55	<0.0001
Cotinine creatinine ratio (CCR) (ng/mg)	598.32 ± 352.13	40.05 ± 27.01	14.14	<0.0001
The number of cigarettes per day	23.50 ± 9.69 (10–60)	—	—	—
Duration of smoking (years)	19.05 ± 4.89 (10–30)	—	—	—

VA: visual acuity; BCVA: best corrected visual acuity; AL: axial length; SE: spherical equivalent; ng: nanogram.

**Table 3:** Retinal nerve fibre layer (RNFL) thickness of the active smokers and passive smokers.

Variable (*μ*m)	Active smokers	Passive smokers	*t* value	*p*
	Mean ± SD	Mean ± SD		
Average RNFL thickness (*μ*m)	104.34 ± 9.12	106.14 ± 8.83	−1.27	0.21
Superior RNFL thickness (*μ*m)	126.84 ± 9.09	133.55 ± 9.91	−4.46	<0.0001
Inferior RNFL thickness (*μ*m)	125.55 ± 11.21	134.73 ± 10.44	−5.36	<0.0001
Temporal RNFL thickness (*μ*m)	73.93 ± 7.09	75.22 ± 4.70	−1.35	0.18
Nasal RNFL thickness (*μ*m)	83.04 ± 10.77	83.41 ± 9.63	−0.23	0.82

**Table 4:** Ganglion cell complex (GCC) thickness of the active smokers and passive smokers.

Variable (*μ*m)	Active smokers	Passive smokers	*t* value	*p*
	Mean ± SD	Mean ± SD		
Upper GCC	98.16 ± 8.27	96.68 ± 5.64	1.32	0.19
Lower GCC	101.55 ± 9.07	101.21 ± 6.26	0.27	0.78
SIN GCC	124.50 ± 7.57	123.94 ± 5.53	0.54	0.59
IIN GCC	123.81 ± 6.83	123.85 ± 5.08	−0.04	0.97
SIT GCC	111.35 ± 5.67	111.61 ± 4.84	−0.32	0.75
IIT GCC	114.77 ± 8.82	114.79 ± 5.95	−0.01	0.99
SON GCC	111.11 ± 11.62	111.63 ± 7.53	−0.33	0.74
ION GCC	115.85 ± 13.49	116.98 ± 9.23	−0.62	0.54
SOT GCC	74.96 ± 5.55	75.35 ± 3.48	−0.53	0.60
IOT GCC	76.35 ± 7.07	77.50 ± 5.80	−1.12	0.26

SIN GCC: superior of inner nasal GCC; SON GCC: superior of outer nasal GCC; IIN GCC: inferior of inner nasal GCC; ION GCC: inferior of outer nasal GCC; SIT GCC: superior of inner temporal GCC; SOT GCC: superior of outer temporal GCC; IIT GCC: inferior of inner temporal GCC; IOT GCC: inferior of outer temporal GCC.

**Table 5:** PERG parameters in the two studied groups.

Variable (*μ*m)	Active smokers	Passive smokers	*t* value	*p*
	Mean ± SD	Mean ± SD		
P50 latency (ms)	52.72 ± 0.52	52.60 ± 0.80	1.19	0.24
N95 latency (ms)	99.94 ± 2.20	98.29 ± 3.22	3.77	0.0002
N35-P50 amplitude (*μ*V)	2.46 ± 0.38	2.54 ± 0.32	−1.49	0.14
P50-N95 amplitude (*μ*V)	3.14 ± 0.41	3.41 ± 0.49	−3.82	0.0002

**Table 6:** Correlations between superior and inferior RNFL thicknesses and different parameters in the active smoker group.

Variables	Active smokers			
	Superior RNFL thickness		Inferior RNFL thickness	
	*r*	*p*	*r*	*p*
Age (years)	0.05	0.66	0.02	0.86
VA (logMAR)	0.01	0.92	0.01	0.92
SE (diopters)	−0.03	0.79	−0.04	0.72
AL (mm)	−0.05	0.66	0.06	0.60
IOP (mmHg)	−0.06	0.60	−0.07	0.54
CCT (mm)	−0.19	0.09	−0.07	0.54
N95 latency (ms)	−0.43	<0.0001	−0.35	<0.0001
P50-N95 amplitude (*μ*V)	0.44	<0.0001	0.40	<0.0001
The number of cigarettes/day	−0.75	<0.0001	0.72	<0.0001
Duration of smoking (years)	−0.03	0.79	−0.08	0.48
Urinary cotinine (ng/ml)	−0.24	<0.0001	−0.31	<0.0001

**Table 7:** Multiple regression analyses assessing different parameters affecting superior RNFL thickness in the active smokers.

	*β*	*B*	*t*	*p* value
Intercept	—	198.93	3.41	0.001
Age (years)	0.11	0.25	1.73	0.09
VA (logMAR)	0.11	10.74	1.74	0.09
SE (diopters)	0.07	1.63	1.12	0.27
AL (mm)	−0.04	−1.23	−0.62	0.54
IOP (mmHg)	−0.04	−0.29	−0.59	0.56
CCT (mm)	−0.03	−0.03	−0.52	0.61
N95 latency (ms)	−0.28	1.15	2.42	0.02
P50-N95 amplitude (*μ*V)	0.31	6.70	2.62	0.01
The number of cigarettes/day	−0.85	−0.79	−13.67	<0.0001
Duration of smoking (years)	0.09	0.17	1.34	0.18
Urinary cotinine (ng/ml)	−0.46	−0.03	−6.49	<0.0001

**Table 8:** Multiple regression analyses assessing different parameters affecting inferior RNFL thickness in the active smokers.

	*β*	*B*	*t*	*p* value
Intercept		145.40	2.03	0.05
Age (years)	0.08	0.25	1.41	0.16
VA (logMAR)	0.11	13.76	1.82	0.07
SE (diopters)	0.09	2.40	1.34	0.19
AL (mm)	−0.02	−0.65	−0.27	0.79
IOP (mmHg)	−0.05	−0.48	−0.80	0.42
CCT (mm)	0.06	0.06	0.97	0.34
N95 latency (ms)	0.20	1.04	1.69	0.09
P50-N95 amplitude (*μ*V)	0.30	8.12	2.48	0.02
The number of cigarettes/day	−0.84	−0.97	−13.68	<0.0001
Duration of smoking (years)	0.07	0.15	0.99	0.33
Urinary cotinine (ng/ml)	−0.53	−0.04	−7.57	<0.0001
